# Human IGF2 Gene Epigenetic and Transcriptional Regulation: At the Core of Developmental Growth and Tumorigenic Behavior

**DOI:** 10.3390/biomedicines11061655

**Published:** 2023-06-07

**Authors:** Pierluigi Scalia, Stephen J. Williams, Yoko Fujita-Yamaguchi

**Affiliations:** 1 ISOPROG-Somatolink EPFP Research Network, Philadelphia, PA 19102, USA, and 93100 Caltanissetta, Italy; 2 Sbarro Cancer Institute for Cancer Research and Molecular Medicine, CST, Biology Department, Temple University, Philadelphia, PA 19122, USA; 3 Arthur Riggs Diabetes & Metabolism Research Institute, Beckman Research Institute of City of Hope, Duarte, CA 91010, USA

**Keywords:** *IGF2*, insulin-like growth factor 2 gene, mRNA transcript, IGF-II, insulin-like growth factor-2 peptide, *p0–p4*: *IGF2* promoters 0–4, TF: transcription factor, DMR, differentially methylated region, CTCF, CCCTC binding factor, CCD, centrally conserved domain, DMD, differentially methylated domain, (IGF2/H19) IG-DMR, intergenic differentially methylated region, ICR1, imprinting control region 1, BWS, Beckwith–Wiedemann syndrome, WT1, Wilms Tumor protein 1, SRS, Silver Russel Syndrome, LOI, loss of imprinting, MOI, maintenance of imprinting, LOM, loss of methylation, GOM, gain of methylation, UPD, uniparental disomy, CNV, copy number variation

## Abstract

Regulation of the human *IGF2* gene displays multiple layers of control, which secures a genetically and epigenetically predetermined gene expression pattern throughout embryonal growth and postnatal life. These predominantly nuclear regulatory mechanisms converge on the function of the *IGF2-H19* gene cluster on Chromosome 11 and ultimately affect *IGF2* gene expression. Deregulation of such control checkpoints leads to the enhancement of *IGF2* gene transcription and/or transcript stabilization, ultimately leading to IGF-II peptide overproduction. This type of anomaly is responsible for the effects observed in terms of both abnormal fetal growth and increased cell proliferation, typically observed in pediatric overgrowth syndromes and cancer. We performed a review of relevant experimental work on the mechanisms affecting the human *IGF2* gene at the epigenetic, transcriptional and transcript regulatory levels. The result of our work, indeed, provides a wider and diversified scenario for *IGF2* gene activation than previously envisioned by shedding new light on its extended regulation. Overall, we focused on the functional integration between the epigenetic and genetic machinery driving its overexpression in overgrowth syndromes and malignancy, independently of the underlying presence of loss of imprinting (LOI). The molecular landscape provided at last strengthens the role of *IGF2* in cancer initiation, progression and malignant phenotype maintenance. Finally, this review suggests potential actionable targets for *IGF2* gene- and regulatory protein target-degradation therapies.

## 1. *IGF2* Gene Regulation at the Promoter and Transcript Level: An Unexploited View

A significant amount of experimental work has been previously produced to address the epigenetic control of the *IGF2* gene among various species, supported by its well-known imprinting associated with DNA methylation [1,2,3]. *IGF2* gene epigenetic regulation has been studied both during mammalian development as well as in *IGF2*-overexpressing syndromes and cancer [4,5,6,7]. Nonetheless, review work specifically addressing human *IGF2* gene regulation at the transcriptional level is missing throughout the literature. This relative lack of recent experimental studies on *IGF2*-specific transcription factors has occurred despite the established biological role of *IGF2* promoter regulation towards *IGF2* transcriptional activation. A number of findings have also addressed *IGF2* transcript regulation via non-coding RNA products (further discussed herein). More recently, among the known *IGF2* (gene) regulatory mechanisms, the role of a family of *IGF2*–mRNA binding proteins (also known as IMPs) has emerged. In light of the increasing number of post-translational mechanisms regulating *IGF2* expression and biological function, especially in cancer, we believe that focusing the current review on the control of *IGF2* at the gene transcription and RNA transcript levels and separating clearly from the post-translational mechanisms is far more important. Accordingly, the present review on human *IGF2* gene regulation focuses, specifically, on integrating the current understanding of *IGF2* gene epigenetic control with the underlying mechanisms occurring at its promoter and transcript (mRNA) levels in cancer and *IGF2*-expression syndromes. Moreover, this review will exclude any discussion of the supplementary non-coding transcripts arising from the *IGF2-H19* gene locus, as no direct modulatory influence on the *IGF2* gene product (RNA or protein) has been conclusively established [8].

## 2. The Human *IGF2* Gene Structure: A Functional Overview

**Figure 1:**
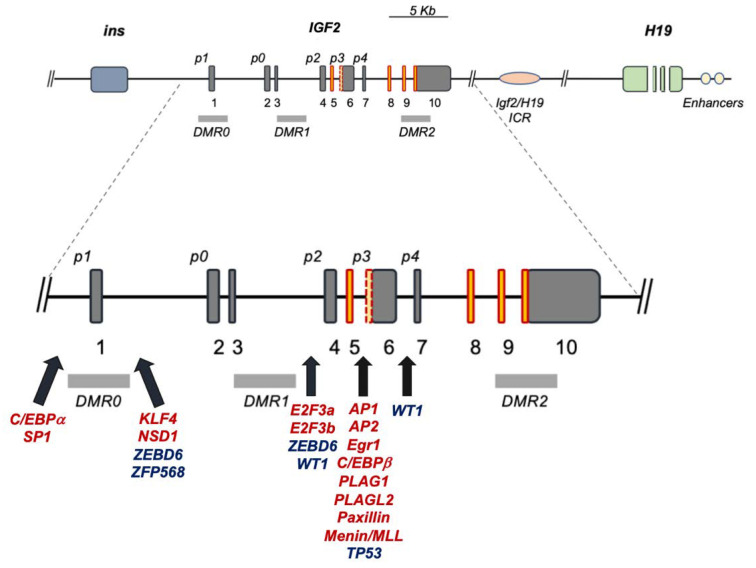
Human *IGF2* gene structure and major transcriptional regulatory sites. Dark-grey boxes: non-coding exons; red-orange boxes: coding exons; red dotted yellow box between *p3* and *p4*: alternatively expressed exon; *p0–4*: *IGF2* promoter regions; light-grey rectangles: DMRs; red transcription factors (TFs): activators; blue TFs: repressors; black solid arrows: IGF2 promoter sites with cited TF binding motifs.

**Figure 2:**
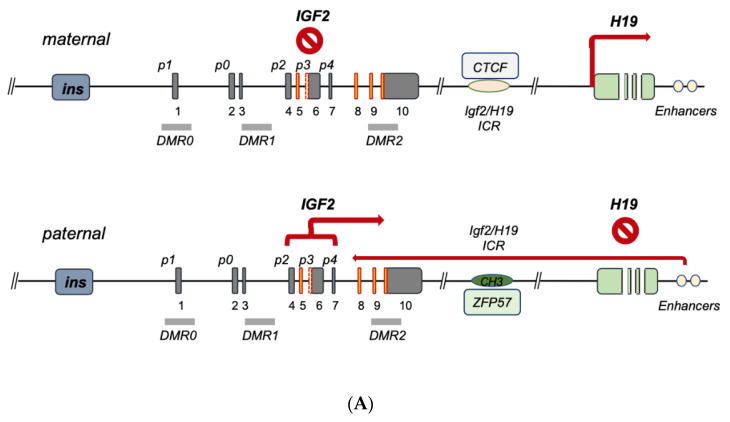

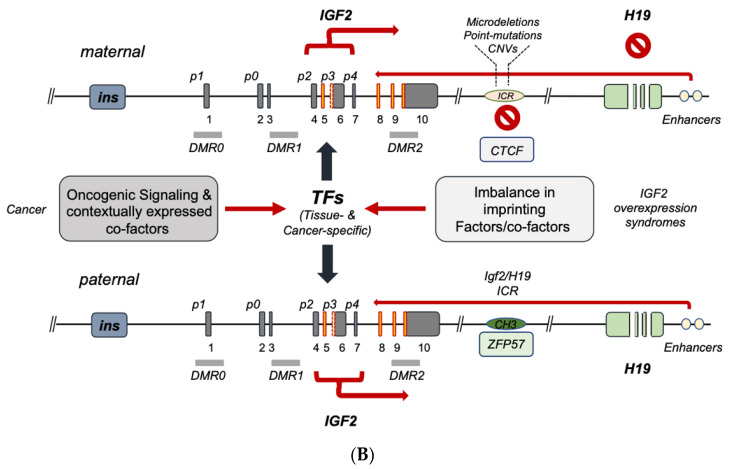
Current model for *IGF2-H19* gene cluster regulation. (**A**) Imprinted *IGF2* gene regulation in embryonal development, post-natal liver and tissues with maintenance of imprinting, MOI. The maternal allele bears a hypomethylated ICR1 allowing for CTFC binding; bound CTCF acts as an insulator by blocking the downstream enhancer effect on *IGF2* which, as a result, is suppressed, while H19 transcription is unleashed by the same CTCF-ICR binding. On the paternal allele, ICR1 is methylated also through ZFP57, which contributes to the maintenance of the methylated status preventing CTCF binding; in absence of CTCF, the downstream enhancers trigger allele (paternal)-specific *IGF2* transcription predominantly through its fetal promoters. (**B**) *IGF2* gene regulation in cancer and *IGF2* overexpression syndromes. In the case of *IGF2* overexpression syndromes (e.g., Beckitt–Wiedemann), *IGF2* expression abnormalities are mostly linked to a number of genetic abnormalities of the maternal *IGF2-H19* ICR (microdeletions, CNVs, point mutations) leading to loss of CTCF binding, imprinting relaxation and biallelic expression of *IGF2*. In case of *IGF2* regulation in cancers, a variety of regulatory modes have been described, which occur either independently or in parallel with the imprinting determining factors converging on each or both the maternal and paternal alleles and ultimately triggering the oncogenic activation of *IGF2* transcription and/or the disruption of *IGF2* transcriptional suppressors. The TF-mediated mechanisms conveyed in (**B**) relate to the potential full biallelic activation, but they can also be observed in monoallelic *IGF2* overexpression.

The human *IGF2* gene occupies the 11p15.5 chromosomal locus, positioned between the insulin gene and the *H19* gene, with which it establishes an imprinted gene cluster (NCBI Ref Seq NG_008849.1) [9] (Figure 1). The *IGF2* gene is composed of 10 exons [10,11,12] whose expression is driven by five promoters (*p0–p4*), differentially activated from embryonal to postnatal life (see Table 2) [13,14,15]. The *IGF2* gene product is a peptidic ligand (IGF-II), which plays a central role in embryonal growth in mammalians [16]. Furthermore, the role of secreted IGF-II autocrine and paracrine effects in tumorigenesis [16] and its growing role towards malignant feature maintenance are well documented (reviewed by Scalia et al. [17]). Interestingly, an alternatively expressed exonic region has been recently described as part of exon 6, and it appears to bear a role in diabetes predisposition (see Figure 1) [18]. For this reason, the understanding of *IGF2* gene expression and transcriptional regulation bears intrinsic high biological and biomedical value. The *IGF2* gene has been widely studied for its epigenetic parental (allele)-specific control. The established evidence demonstrates that in the majority of adult tissues, *IGF2* is exclusively expressed by the paternal (methylated) allele due to its imprinting on the maternal (hypomethylated) allele, which is silenced as a result of its hypo-methylation status. In particular, the *IGF2* promoter-specific differentially methylated regions (DMRs 0, 1, 2) partially overlap the *IGF2* intronic and exonic sequences, along with the DMR known as “inter-genic- or IG-DMR” or Imprinting Center Region 1, ICR1. This region is located between the *IGF2* and *H19* genes coding regions and the *IGF2* enhancer region downstream from *H19*, cumulatively establishing a phylogenetically conserved gene cluster acting as an epigenetic switch [19,20,21]. IG-DMR is an allele-dependent DMR (ICR1) containing the binding motif for the epigenetic master regulator CTCF, which, along with the PRC2 complex components (discussed in Section 2 and summarized in Table 1). The CTCF–PRC2 complex binds the maternal hypomethylated ICR and insulates the *IGF2* promoters [22,23]. On the contrary, the paternal ICR1, being prevented from CTCF binding as a consequence of ICR1 methylation status, results in a fully receptive effect of the enhancer regions, thereby displaying the classic monoallelic expression of the human imprinted *IGF2* locus. This is graphically summarized in Figure 2A. A parallel promoter activation pattern for *IGF2* expression in fetal growth, compared to the postnatal and adult phases, includes the promoter usage switching from the imprinted “fetal” (*p2–p4*) and “placental” (*p0*) promoters to the adult (p1) promoter [9]. Indeed, both cumulative and recent findings display a more diversified landscape of IGF2 regulation, extending beyond the previously known abnormalities linked to either (a) epigenetic deregulation or (b) allelic (uniparental) disomy, both of which are described in *IGF2* overgrowth syndromes [24]. Therefore, it is more correct to state that *IGF2* displays developmental-, tissue- and disease-specific gene expression patterns, as discussed further in the present review. It is important to note that, regardless of which promoter region drives *IGF2* primary transcript, the translation of all transcripts leads to a single 180aa pre-pro-peptide, which is further processed to a 67 KDa peptide in normal somatic cells. An exception to the above IGF-II post-translational peptidic maturation/processing is typically observed in cancer, resulting in the production of a glycosylated high-molecular-weight peptide variant, which bears additional biological advantage for cancer progression, as reviewed elsewhere [17].

## 3. *IGF2* Gene Regulation during Development and *IGF2* Overexpression Syndromes

Seminal studies have shown the importance of genomic imprinting for the *IGF2* gene and the entire *IGF2-H19* gene cluster (reviewed in [9]). As discussed in the previous section, ICR1 differentially methylated status affects the binding of epigenetic master-regulator CTCF to unmethylated ICR1 motifs acting as an insulator [2,22,25,26,27,28]. More recently, the role of the imprinting factor ZFP57 on the methylation maintenance status of the paternal allele has been shown [29]. The above control model for *IGF2* imprinting on the maternal and paternal alleles is schematically summarized in Figure 2A.

The term ‘*IGF2* overexpression syndromes’ relates to a variety of genetic abnormalities sharing the phenotype reported by Beckitt–Wiedemann to describe the resulting pediatric syndrome. A majority of the overgrowth symptoms in these subjects are secondary to the high levels of IGF-II produced at the embryonal and postnatal levels. This overproduction is mostly linked to the biallelic expression of *IGF2* as a result of the imprinting relaxation of the maternal allele. A recent analysis of the genetic abnormalities in these subjects [24], leading to increased *IGF2* activation, allows one to functionally classify the currently known *IGF2* gene expression defects into two types, namely, (a) ICR defects causing the above relaxation on the maternal allele (via microdeletions and/or to DMR point mutations) [23,30,31,32], and (b) quantitative defects affecting the overall paternal gene cluster as a result of either uniparental disomy (UPD) or copy number variations (CNVs) [30,33,34]. An additional layer of control on *IGF2* imprinting disclosed by recent studies relates to allele/DMR-specific factors acting as intrinsic-enabling factors and/or acting in synergy with CTCF on the maternal ICR [35]. Among these are Sox2/Oct4 [36,37], SUZ12 [38] and Vigilin [39], whose contextual functions as imprinting factors are governed by histone post-translational modifications, as evidenced by studies confirming their role in affecting both general and specific *IGF2* imprinting. In particular, histone acetylation has been recognized since the late 1990s as a regulator of *IGF2* imprinting, as shown by the ability of histone deacetylase inhibitors to cause *IGF2* biallelic expression [40]. Even more detailed is the demonstration of the key role of H3K27 histone methylation for the proper maintenance of the maternal imprinting status via its effects on (a) the *IGF2-H19* cluster loop conformation and (b) the DNA protein complex formation on the imprinted maternal allele [41]. In fact, in those cells with loss of imprinting (LOI), H3K27 demethylation leads to loss of the CTCF-orchestrated intrachromosomal loop between the *IGF2* promoters and the ICR. The H3K27 methylation-free *IGF2* promoters appear to become activated similarly to the paternal promoters, leading to biallelic expression. Noteworthily, SUZ12 has been shown to play a key role in the maintenance of the hypermethylation status of H3K27 by EZH2 since, in the absence of SUZ12, the PRC2 cannot be recruited to the maternal *IGF2* promoter where this methylation takes place in order to induce the imprinting loop conformation [41]. The chromatin conformation at the *IGF2-H19* cluster locus has been found to be essential for proper *IGF2* expression, and this higher-order chromatin organization function is mediated by Cohesin [42]. Altogether, these studies point at a wider molecular network for the allele-specific control of *IGF2* imprinting and offer additional potential mechanisms of dysregulation that could be responsible for those, yet unaccounted, molecular defects, leading to *IGF2* increased transcription underlying the pathologic conditions discussed herein. A graphic summary of the human *IGF2-H19* cluster regulation focusing on the latest landscape provided by the reviewed literature is conveyed in Figure 2 and Table 1.

**Table 1:** Factors binding *IGF2-H19* ICR1 and affecting IGF2 imprinting status.

Imprinting Factor	Key Feature	Reference(s)
*CTCF*	binds maternal ICR and insulates *IGF2-p* activity	[35]
*Cohesin*	Cohesin is required for chromatin function at the *H19/IGF2* locus	[42]
*EZH2*	CH3-transferase component of PRC2	[41]
*SUZ12*	PRC2 component enabling ICR imprinting	[38]
*Sox2/Oct3–4*	CTCF-like effect	[36]
*Vigilin*	ICR imprinting effect via CTCF binding	[39]
*ZFP57*	Binds paternal ICR and maintains methylated status	[29]

## 4. *IGF2* Gene Transcriptional Control in Cancer

A number of studies focusing on the role of *IGF2* gene methylation and promoter usage in cancer have established the importance of *IGF2* LOI status [43,44,45,46]. Nonetheless, the mechanistic relationship between promoter usage, both under monoallelic (under maintenance of imprinting, MOI) and biallelic status (caused by LOI), and the observed total *IGF2* expression pattern/levels in cancer remains an active area of investigation. Indeed, a number of studies have shown a predominant activation of *IGF2* fetal promoters (*p2–p4*) in a variety of cancers displaying *IGF2* increased expression levels, with variable uncoupling of DMR_0–2_ methylation, along with monoallelic *IGF2* and/or *H19* expression [47,48,49,50,51,52,53,54,55,56]. In this context, it is important to stress that promoter usage and transcriptional activity are directly dependent on the involved transcription machinery, which is affected, in its turn, by the contextual transcriptional co-activator and co-repressor effects (also provided by the underlying *IGF2* epigenetic protein–DNA interactions). For this reason, unlike other published review works (to date), we here discuss the promoter usage in terms of underlying transcriptional machinery (see Section 4.1 and Section 4.2 below). In addition to the protein/DNA-driven control layer (or lack of control) of *IGF2* gene expression, it is important to add the regulation layer provided by the RNA transcript control (Section 4.4 and Section 4.5 below). This type of integrated approach to study *IGF2* gene regulation both in *IGF2* expression syndromes and in cancer, according to the authors of the present work, is essential in order to move the field beyond the historical (and still ongoing) compartmentalized approach to *IGF2* gene studies. A graphic summary of our current understanding of the regulation of the human *IGF2* gene, spanning from *IGF2* expression syndromes to cancer (overlapping in vivo), is provided in Figure 2B and Table 2.

**Table 2:** *IGF2* promoter usage in physiology and disease.

Promoter Usage	Imprinting Control	Reference(s)
*IGF2-p0*	Not imprinted Mostly active in fetal placenta	[15]
*IGF2-p1*	Not imprinted—mostly active in postnatal Liver	[57,58]
*IGF2-p2*	Imprinted- Mostly active during Fetal growth	[59]
*IGF2-p3 &* *IGF2-p3/p4* (*)	Imprinted- Mostly active during Fetal growth, Widely reactivated in cancer	[51,57,58,60]

(*) *P3* and *P4* are indicated together due to shared binding motifs often causing consensual activation.

### 4.1. Transcription Factors Regulating IGF2 through Its Fetal Promoters

*IGF2 p2–p4* are all imprinted promoters [13]. Therefore, as discussed for the IGF2 overexpression syndromes, the relaxation of their imprinting status can play a promoting role in IGF2 activation, as confirmed in a set of cancers displaying LOI. Nonetheless, to date, a few studies have shed full light on the molecular mechanisms and dynamic modifications in DNA methylation occurring at the *IGF2* DMRs partially overlapping with the *IGF2* main promoters located upstream of ICR1 (responsible for the *IGF2* imprinting status). This is important given that their methylation status plays a direct role in the transcriptional activation of the underlying *IGF2* promoters in cancer.

*AP-1*. AP-1 is a collective term referring to dimeric transcription factors sharing high affinity and activating ability for the AP-1 binding site when present at a promoter DNA level. The AP-1 TF dimer components are members of the Jun, Fos and ATF DNA-binding protein families (reviewed in [61]). The IGF2 gene has been shown to contain AP-1 binding motifs in its promoter *p3*, and mammalian *IGF2* transcription is activated by AP-1 [62]. Nonetheless, the exact composition of the AP dimers involved in the *IGF2* gene regulation under the various physiological and pathological contexts remains to be clarified.

*AP-2*. AP-2 refers to dimeric transcription factors of the homonymous family, which binds to a CG-rich DNA binding motif and is involved in cellular proliferation and suppression of terminal differentiation in embryonic development [63]. AP-2 has been shown to activate *IGF2* transcription via *IGF2-p3*, which has been found to contain four AP-2 binding motifs [64].

*C/EBPβ*. C/EBP’s role in *IGF2-p1* activation in human liver cells and tissues has been known since the early 1990s [65]. More recently, it was shown that 2,3,7,8-Tetrachlorodibenzo-p-dioxin (TCDD) was able to trigger the onset of hepatoma in a rodent model via specific induction of *IGF2* transcription, concomitant with the increase in C/EBPβ promoter activity. The identification of a specific C/EBPβ binding motif within the *IGF2-p3* promoter, whose activity increased upon TCDD treatment, further supports the role of this C/EBP paralog in *IGF2*-driven tumorigenesis [66]. This finding assumes particular interest in *IGF2*-driven cancer in light of the mechanistic demonstration of the dimeric requirement of C/EBPs towards gene target transcriptional activation, similarly to other bZIP family of DNA binding proteins (reviewed in [67]). The potential cooperative effects observed between C/EBPβ and SP1 on other promoter types [68] have also been found to play a role towards switching promoter binding affinities towards modulating gene expression, in this case allowing C/EBPβ to gain a permissive role over SP1 activation of *IGF2-p1*, as further discussed under Sp1 below.

*Egr1 and Egr2*. Egr1, also known as Krox24, is a transcription factor of the zinc-finger family binding *IGF2-p3*, as part of a constitutive response pathway to hypoxia [69]. Egr2, also known as Krox20, is a putative activator of *IGF2* gene transcription based on the identified binding site on *IGF2-p3* [10]. Interestingly, the IGF-II signal is also involved in HIF-1α upregulation [70], suggesting the establishment of a positive feedback mechanism requiring *IGF2* local gene upregulation under hypoxic conditions [71]. This type of mechanism is particularly important in solid cancer progression, which is characterized by hypoxia during the early phases of tridimensional growth and as part of the observed cancer angiogenic switch (reviewed in [71]).

*E2F3*. E2F3 has been shown to play a key role in driving *IGF2* expression in postnatal cells and tissues, and the decline in E2F3 levels correlates with the reduction in age-related *IGF2* levels, both in humans and in other mammalians [59]. Furthermore, E2F3-overexpressing cancer cell lines display increased *IGF2* expression, which can provide an LOI-independent mechanism for *IGF2* regulation in cancer. For such reasons, inclusion of E2F3 in expression screening panels to identify and properly treat *IGF2*-driven cancers in adults has a proper rationale.

*Menin/MLL*. As previously discussed in relation to the role of H3K27 methylation, the Menin/MLL complex has been shown to play a role in the activation of *IGF2 p3* and p4 promoters in hepatocellular carcinoma [72]. As shown by the study, the role of the Menin/MLL complex provides another mechanism to overcome the imprinting-promoting status of H3K27 methylation on the maternal allele, ultimately allowing for biallelic *IGF2* expression and potentiation of the IGF-II oncogenic signal in liver cancer.

*PLAG1/PLAGL2* [73,74,75,76]. PLAG1, along with PLAG-like protein 2 (PLAGL2), belongs to a subfamily of zinc-finger proteins [74]. PLAG1 was first isolated from salivary gland adenomas [73], while PLAGL2 was isolated in hepatoblastomas. PLAG1 expression mirrors that of IGF-II, with high-level expression during fetal development and a substantial decrease after birth. Both PLAG1 and PLAGL2 overexpression in cultured cells triggers *IGF2* gene expression and induces cancer-promoting effects, including transformation [74]. Five putative PLAG1 binding sites have been identified in *IGF2-p3* and IGF2 transcript size described in such tumors (6 Kb), corresponding to *IGF2-p3*. This further strengthens the claims that *IGF2* is a PLAG1/PLAGL2 transcriptionally activated target. The finding that PLAG1 is overexpressed and induces *IGF2-p3* activation also in hepatoblastoma cell lines [75] suggests that PLAG1 and PLAGL2 may play a wider role in *IGF2* overexpression in cancer. Equally important is the parallel observation that cancers overexpressing PLAG1 did not show *IGF2* LOI [75], supporting the latest view that overexpression of specific *IGF2* promoters targeting transcription factors constitutes an important, still unexploited, LOI-independent mechanism for *IGF2*-mediated tumorigenesis.

*TP53*. The suppressor effect of TP53 on *IGF2* gene activation at the level of its *p3* promoter has been established since the mid-1990s [77]. This discovery has provided a rationale for the inverse relationship demonstrated in cancer between *IGF2* expression and TP53 status [78], reinforcing the role of IGF-II as a bona fide oncogenic factor in a variety of tumors [17,79]. The role of the TP53/*IGF2* axis towards IGF-mediated effects in cancer have been discussed elsewhere [80].

*ZBED6*. The finding of a mutation on the third *IGF2* intronic region in pigs with an overgrowth phenotype [81] interfering with ZBED6 binding to a conserved *IGF2* promoter site among placental mammals [82] places this TF among the negative regulators of *IGF2* expression. As discussed in the next section, despite its binding site location suggesting a potential effect on *p3,* it actually affects *IGF2* expression via *p2* and *p1* at the postnatal level [82].

*WT1*. Wilms Tumor protein 1 (WT1) is a zinc-finger domain-containing protein [83]. WT1 suppresses *IGF2* gene expression [84] through physical interaction to the same DNA binding motif used by Egr1 on *IGF2-p3* [85], specifically at the level of its *p3* and *p4* promoters [86,87]. However, the demonstration that WT1 also binds with high affinity to the corresponding transcribed motif and inhibits translation of the *IGF2* RNA transcript, along with the parallel finding that its nuclear localization overlaps with RNAse-sensitive rather than DNAse-sensitive areas [88], offers a wider scenario for the WT1 suppressing capability of *IGF2* expression in vivo. Indeed, in Wilms tumors, in which WT1 is biallelically mutated or deleted, Egr1 is upregulated, offering a dual synergistic mechanism for *IGF2* increased transcriptional activation [89]. In addition, *IGF2* transcription can be differentially affected by the various WT1 isoforms, which are expressed in a tissue- and cancer-specific context. In fact, two isoforms of WT1 have been described, differing in three amino acids (KTS) at the 5′ junction [90,91]. The full-length isoform (+KTS) is the form most widely expressed and studied, and it is responsible for the observed suppressive effect on *IGF2-p3.* Interestingly, the less common but contextually expressed −KTS WT1 isoform displays gene activating ability through the same promoter, independently of TP53 status [92]. Altogether, these findings suggest that isoform-specific transcriptomic profiling is a critical strategy towards developing molecular therapeutics specifically aimed to target gene expression mechanisms.

### 4.2. Transcription Factors and Other Co-Factors Regulating IGF2 via Its Placental (p0) and Adult (p1) Promoters

Promoters *p1/p0* (adult and placental, respectively) are found activated after birth at variable levels and co-expression with transcripts generated by *p2–p4* in those tissues which have retained fetal promoter activity. Indeed, a number of studies have shown that the human *IGF2* promoter usage in cancer switches from monoallelic *p2–p4 IGF2* control to biallelic *IGF2* expression under *p1* control during tumorigenesis [43]. Another study looking at the relationship between LOI and promoter usage in cervical carcinoma [52] found an exclusive usage of *p1* in tumor tissues with underlying *IGF2* LOI but not in those tumors with maintenance of *IGF2* imprinting (MOI). This suggests that promoter usage (both for *IGF2* and other cancer-driving genes) is cancer-type-specific and/or it may reflect the specific tumorigenic stage (specifically, early/partially differentiated versus advanced/fully dedifferentiated). In the cited study, the overall *IGF2* transcript in LOI tumors was increased along with markedly reduced *H19* levels compared to normal tissues, confirming a general hallmark of the *IGF2-H19* cluster expression pattern used to differentiate pathologic from normal conditions, since under normal conditions, the observed *IGF2* and *H19* expression levels are comparable. A previous study on cervical carcinoma found similar uncoupling of *H19* and *IGF2* expression along with biallelic hypomethylation of *H19* [48]. Other studies displaying a variable level of *p1* usage under LOI have been reported in Wilms tumors [49] and hepatoblastoma [93]. This further supports the view that additional mechanisms for *IGF2* gene activation take place during the tumorigenic process to act in parallel or independently from the imprinting molecular machinery. Such mechanisms aim to regain usage of favorable *IGF2* promoter activities on a cancer-type and cell-status basis. The specific transcription factors interacting with the *IGF2* “placental” (*p0*) and “adult” (*p1*) promoters cause *IGF2* transcriptional activation.

*C/EBPα*. As mentioned earlier, C/EBPα has been described to be an *IGF2-p1* activating factor in postnatal and adult mammalian and human liver [94,95]. More recent findings (reviewed in Lekstrom-Himes et al. [67]) suggest a dominant role of C/EBPα compared to C/EBPβ paralog over the choice of the promoter usage. This supports the idea that both C/EBPα homodimers as well as C/EBPα/β heterodimers would use *p1* to increase *IGF2* transcriptional levels while only C/EBPβ homodimers could be able to trigger *IGF2-p3* transcription. Additional studies are needed to clarify the role of C/EPB heterodimers towards *IGF2* expression in cancer. Interestingly, C/EBPα along with its beta paralog may also contribute to Sp1-driven activation of *IGF2-p1,* as further discussed herein.

*KLF4*. KLF4 is a member of the Kruppel-like transcription factor family, playing a cancer-type-specific role as an activator of epithelial genes involved in differentiation and a repressor of mesenchymal genes involved in EMT and metastasis [96]. On the other hand, KLF4 has been found to have repressor activity towards tumor suppressors such as TP53, ultimately conferring to it a contextual oncogenic effect [97]. These latest considerations could be relevant in light of the finding that a KLF4 binding motif has been identified within *IGF2*-DMR_0_ and that KLF4 has been found to activate *IGF2-p0* in a DMR_0_ hypomethylation- and H3K27-methylation-dependent modes. This is independent of the underlying LOI and ICR1 methylation status [98]. Based on these reviewed findings, KLF4 plays a contextual activation role on *IGF2* transcription both directly [98] as well as via suppression of TP53 [97]. Its mechanistic involvement in *IGF2*-driven cancers requires further attention.

*NSD1*. Interestingly, a study conducted on an overgrowth syndrome characterized by NSD1 deficiency [99], a molecular defect found in a subset of BWS, demonstrated the role of NSD1 as a specific co-activator for a novel enhancer in DMR0 affecting *IGF2-p0* transcriptional activity in a cell-type-specific manner. This finding raises the question of a potential role of NSD1 and its demethylase activity in the *IGF2*-driven tumorigenic process.

*Sp1*. A finding bearing plenty of biological value for its mechanistic implication relates to the identification of an Sp1 binding motif on *IGF2-p1* [100]. The results of this study have shown (a) the requirement for specific positioning and sequence specificity of the Sp1 binding motif within *IGF2-p1*, and (b) the cooperative role of C/EBPα and C/EBPβ through binding to the close C/EBP site towards *IGF2-p1* activation by Sp1. The same type of cooperative control by Sp1 and C/EBPs has been observed on other promoters [68]. This is particularly important given the inability of C/EBPβ to bind to *p1* as a homodimer supporting that the co-expression of Sp1 and C/EBP1β may play a role in allowing for efficient *IGF2* expression via its *p1* promoter beyond the potential role of C/EBPβ homodimers on *IGF2-p3*.

*ZBED6*. A recent study identified the transcription factor ZBED6 as a major regulator of postnatal *IGF2* expression in mammalians acting as a strong repressor of *IGF2* in a number of adult tissues through its specific effects on the activity of *IGF2 p1(adult)-p2 (fetal)* promoters [82]. This study disclosed the important role of the ZBED6–*IGF2* axis in regulating *IGF2* expression, muscle growth and the growth of internal organs in placental mammals. The specific role of this axis in cancer promotion in adult tissues remains to be elucidated.

*ZFP568*. Another member of the Kruppel zinc-finger family, ZFP568, was found to repress IGF2 transcription at the level of its *p0* promoter in the placenta [101]. The actual role of this TF in affecting *IGF2*-driven tumorigenesis is still to be determined.

The reviewed TFs and their binding sites within the *IGF2* gene promoters are summarized in Figure 1 and listed in Table 3.

An important area of current and future investigation towards understanding *IGF2* gene activation by both transcription factors and imprinting factors (co-activators and/or co-repressors) relates to their ability to bind and activate preferentially or exclusively unmethylated rather than methylated binding sites, and vice versa. This potential scenario is even more relevant for those *IGF2*-regulating TFs, for which DNA binding motifs contain CpG islands, such as for KLF4, ZPF568 [87] and C/EBP (reviewed by Blatter et al., 2013) [103].

### 4.3. Transcription Factors and Co-Factors Affecting IGF2 Transcription through Interactions Outside the IGF2 Direct Regulatory Cluster

This category is expected to grow substantially in the future. To date, a few examples we found in the literature can be useful to grasp the potential of this category. One is provided by the muscle-tissue-specific TF MyoD, which has been found to interact with a site on *H19* to actually promote its activation while inhibiting *IGF2* activity during differentiation of specific smooth muscle districts such as in mouse diaphragm formation [104]. The other example, which may actually have a greater effect on *IGF2* cancer-related effects, relates to Paxillin, a focal adhesion protein found to bear unexploited nuclear functions as a chromatin interacting co-factor, specifically affecting a number of genes; in particular, Paxillin has been found to activate *IGF2* transcriptional activation by stimulating the interaction between the enhancer region and the *IGF2* promoters while restraining the interaction between the enhancer and *H19* via downregulation of it gene [102]. Such an effect seems to be mediated through a protein complex formation with Cohesin, allowing it to mediate long-range chromosomal looping. Such findings suggest that Paxillin may play a supporting role in proliferation and fetal development through *IGF2* gene upregulation.

### 4.4. Regulation of IGF2 Gene Expression via mRNA Stabilization and Beyond

Another level of regulation of *IGF2* gene expression occurs through specific binding of its RNA transcript at the level of the 5′UTR by a family of RNA binding proteins that are known as IGF2BP-1, IGF2BP-2 and IGFBP-3, and by their acronyms IMP-1, IMP-2 and IMP-3 (reviewed by Bell et al. [105]). They are a class of oncofetal proteins preferentially expressed during development as well as in a variety of cancer cell types and are able to bind a high number of RNAs estimated in the range of hundreds, although an exact number has not yet been determined. IGF2BP2 is also expressed in two isoforms of 58 kDa and 65kDa [106]. All of them have been demonstrated to bear cancer-promoting functions [107,108,109,110,111]. However, while all of them can bind the *IGF2* transcript, IGF2BP2 and IGF2BP-3 have been shown to specifically enhance *IGF2* expression [112,113]. In particular, the IGF2BP2-driven *IGF2*-enhancing effect in cancer seems to be synergistically exerted by the parallel suppression of *IGF2* signal inhibitors [110]. Post-translational modification of IGF2BPs by upstream-initiated signals has been already shown for some of them [112,114] and is expected to shed light on their cellular regulation and functional network. IGF2BPs are the object of an active area of investigation given their cancer-promoting effects extending beyond the ones mediated via *IGF2* transcript binding [115].

### 4.5. Non-Coding RNA-Mediated Regulation of IGF2 Gene Expression

It has become increasingly apparent that multiple non-coding RNAs control *IGF2* transcript expression. Nctc1 is a cell-/tissue-specific long-non-coding (lnc) RNA co-regulated by the *IGF2*-H19 cluster found to interact on their allelic shared enhancer [116]. miR-30e was shown to suppress *IGF2* in mesenchymal and aortic smooth muscle cells, decreasing proliferation and increasing differentiation [117]. *IGF2* was identified as a major target of miR-125b in the search for skeletal muscle regulators [118]. Interestingly, miR-125b and miR-100 have been found to confer stem-like features to hepatocellular carcinoma cells [119]. A recent study in Wilms tumor patients’ derived cell lines found *IGF2* to be downregulated by miR-155-5p via direct binding to its 3’ untranslated region [120]. In these cells, miR-155-5p upregulation and *IGF2* knockdown suppressed cell proliferation, migration and invasion and induced cell apoptosis. mir185-5p has been found to bind both NEAT1 and *IGF2* transcripts, and it upregulates *IGF2* while enhancing migration and invasion in colon cancer cells [121]. *IGF2* is a direct target of miR-210 in human retinal endothelial cells [122]. miR-223 has been found to suppress *IGF2* and Zeb1 in myoblasts [123]. miR-483-5p, a microRNA (miRNA) overexpressed in primary Wilms’ tumors, has been found to upregulate *IGF2* mRNA through enhancement of its transcriptional levels [124]. The study also showed its ectopic expression in IGF-II-dependent sarcoma cells and its direct correlation to the increased tumorigenesis in vivo. miR-483-5p is one of the two miRNAs (483-5p and 483-3p) generated at the same locus and embedded within the *IGF2* gene. A study found miR-483-5p to be expressed in esophageal squamous cell carcinoma (ESCC), with low levels of *IGF2* promoter methylation (associated with increased *IGF2* expression) and proposed a permissive level for *IGF2* promoter methylation towards the observed miR-483-5p tumor suppressing effects [125]. Interestingly, in hepatocarcinoma cells, miR-493-5p displayed anti-tumor activity by inhibiting the *IGF2*-derived intronic miR-483-3p, the expression of which correlates with *IGF2*-LOI in liver cancer cells [126]. MiR-543 has been found to inhibit ovarian cancer cell proliferation by suppressing *IGF2*, and this effect would reflect its levels in exosomes [127]. Another miRNA contributing to the epigenetic regulation of *IGF2* in cancer is miR-615-5p, which has been found to bear tumor-suppressing abilities by inhibiting proliferation, migration and invasion in pancreatic ductal adenocarcinoma (PDAC) primary cell lines [128]. The suppressing role of miR-615-5p has also been studied in endothelial cells where the *IGF2* block affects the VEGF-AKT/eNOS signaling, decreasing angiogenesis [129]. Given the demonstration by the same study of parallel inhibition of JunB, future studies will have to quantify the role of *IGF2* suppression on the whole tumor-suppressing activities of this miRNA. More recently, the demonstration that the transcription factor pleomorphic adenoma gene 1 (PLAG1), overexpressed in Wilms tumors with mutations in microRNA processing genes, is a target gene of microRNA strengthens the role of this class of non-coding transcripts in the regulation of the *IGF2* gene in cancer [130]. miR-491-5p, which plays a role in cancer initiation and progression, displayed tumor-suppressing activity linked to targeting of *IGF2* in a study conducted in cell lines, tissues and plasma from CRC patients [46]. Hypoxia-inhibited miR-4521 has been found to inactivate the AKT/GSK3b/SNAIL pathway by targeting *IGF2* and FOXM1 and suppressing gastric carcinoma [131]. miR-6759-5p was found to target *IGF2* and is suppressed by a long-non-coding RNA (lnc-MCEI), which stimulates *IGF2*-mediated chemosensitivity in esophageal squamous cell carcinoma (ESCC) [132]. This evidence further confirms the widespread role of non-coding regulatory RNA transcripts in gene activity regulation and sheds additional light on the parallel positive and negative feedback affecting the *IGF2* gene. The non-coding RNAs found involved in *IGF2* regulation to date are listed in Table 4 below.

### 4.6. IGF2-p3 Functional Block: A Valuable Targeting Strategy for Cancer Gene Therapy?

Given the cumulative evidence displaying such wider preference for *IGF2-p3* by established oncogenic TFs, a new class of gene therapies targeting *IGF2-p3*’s function may bear a distinctive advantage in cancer therapeutics. Such pharmacological strategies may consider either a direct (e.g., gene editing) or an indirect (protein degradation of key oncogenic TFs) targeting mode and be classified under the proposed category of “*IGF2-p3* blockers” to indicate their planned or otherwise established mode of action. Surely, the technological advancement in effective cellular targeting of previously undruggable gene products bearing non-enzymatic classic activity, such as transcription factors using protein-targeted degradation strategies (e.g., PROTAC and related technologies) [141,142,143], further supports the design and adoption of this new class of smart therapeutics. 

## 5. Conclusions and Perspectives

A growing set of studies provide a scenario in which modifications in the methylation status (both general and promoter-specific) of the *IGF2* gene (independently of its biallelic expression as result of LOI) do not fully explain both the levels and patterns of *IGF2* gene product expression observed in cancer. This is in line with the recent mechanistic findings disclosing additional layers of *IGF2* transcriptional control. Among these controls are the mechanisms regulating *IGF2* RNA transcripts discussed herein. The contextual and/or parallel role of tissue- and cancer-specific transcription factor expression (and potential underlying isoform or mutant variants) has also become evident as a crucial factor for the biological enhancement in those cancer subsets with either LOI or MOI of the *IGF2* gene. Overall, despite more mechanistic studies being expected to emerge in the future, the scientific work generated to date suggests a more complex and highly integrated scenario underlying the expression and transcriptional activation of the human *IGF2* gene. This clearly suggests that more integrated and multilayer analysis of the *IGF2* gene control should be taken into consideration for the design of future studies pointing at the characterization and modulation of the *IGF2* gene in cancer. The new research approach proposed and integrated in this review should move beyond the simple LOI and promoter usage evaluation and include the detection/analysis of the underlying components of the transcriptional machinery on a case-by-case basis. This would eventually allow us to design new strategies with more accurate diagnostic and therapeutic potential.

## Figures and Tables

**Table 3:** Transcription factors associated with human/mammalian *IGF2* gene regulation.

Transcription Factor	TF Motif(s) in hIGF2 Promoter	Effect on IGF2 Gene Transcription	Reference(s)
*AP1*	*p3*	Activator	[62]
*AP2*	*p3*	Activator	[64]
*C/EBPα*	*p1*	Activator	[94]
*C/EBPβ*	*p3*	Activator	[66]
*E2F3*	*p2*	Activator	[59]
*Egr1*(*Krox24*)	*p3*	Activator	[69]
*Egr2*(*Krox20*)	*p3*	Activator	[10]
*KLF4*	*p0*	Activator	[98]
*Menin/MLL*	*p3*	Activator	[72]
*NSD1*	*p0*	Activator	[99]
*PLAG1*	*p3*	Activator	[76]
*Paxillin*	*p3*	Activator	[102]
*TP53*	*p3*	Repressor	[72]
*SP1*	*p1*	Activator	[100]
*ZBED6*	*p1-p2*	Repressor	[82]
*ZPF568*	*p0*	Repressor	[101]
*WT1*	*p2/p4*	Repressor	[85,87]

**Table 4:** Non-coding RNAs affecting *IGF2* gene expression.

miRNA	Features	References
*Nctc1*	Coregulated with *IGF2* at muscle enhancer	[116]
*Let-7*	Suppresses IMP1-3 and their oncogenic potential	[133]
*Let-7a/a-3*	Let7a-3 hypermethylation associated with low *IGF2* in ovarian and Breast cancer	[134]
*Let-7i*	Suppresses IGF2BP2-3 in HCC	[135]
*miR-23a-5p*	Suppresses *IGF2* and its inhibition by circular non-coding RNA (100084) stimulates HCC	[136]
*miR-30e*	Suppressed *IGF2* in mesenchymal cells	[117]
*miR-100*	Confers stem cell features to HCC	[119]
*miR-125b*	Suppresses *IGF2* in skeletal muscle Confers stem cell features to HCC	[118]
*miR-155-5p*	Suppresses *IGF2* and PI3K-AKT in WT	[120]
*miR-185-5p*	Mediates NEAT1 upregulation of *IGF2* in CRC	[121]
*miR-210*	Suppresses *IGF2* in HRECs	[122]
*miR-223*	Suppresses *IGF2* and ZEB1 in myoblasts	[123]
*miR-483-3p*	Is co-regulated and over-expressed in WT, CRC, Breast ca, and HCC	[137]
*miR-483-5p*	Overexpressed in WT/ Enhances *IGF2* Increased in low methylated *IGF2* promoter ESCC	[124]
*miR-486-5p*	Upregulate *IGF2*/βCatenin axis effects in CRC by suppression of PLAG2	[138]
*miR-491-5p*	Suppresses *IGF2* in CRC	[46]
*miR-493-5p*	Suppresses miR-483-5p in HCC/ inhibits *IGF2*	[126]
*miR-543*	Suppresses *IGF2* and ovarian ca cells proliferation	[127]
*miR-4521*	Suppresses *IGF2* and FOXM in gastric ca	[131]
*miR-615-3p*	Inhibits *IGF2* in NSLC	[139]
*miR-615-5p*	Suppresses *IGF2* in Human PDAC- Inhibits angiogenesis by targeting IGF2 in ECs	[128]
*miR-1275*	Suppresses IGF2BP1-3 and inhibits HCC malignant growth	[140]
*miR-6759-5p*	Suppresses *IGF2* and mediates the competing effects of lnc-MCEI in ESCC	[132]

## Data Availability

No new data have been generated.
